# TSPAN7 Exerts Anti-Tumor Effects in Bladder Cancer Through the PTEN/PI3K/AKT Pathway

**DOI:** 10.3389/fonc.2020.613869

**Published:** 2021-01-08

**Authors:** Xi Yu, Shenglan Li, Mingrui Pang, Yang Du, Tao Xu, Tao Bai, Kang Yang, Juncheng Hu, Shaoming Zhu, Lei Wang, Xiuheng Liu

**Affiliations:** ^1^ Department of Urology, Renmin Hospital of Wuhan University, Wuhan, China; ^2^ Department of Radiography, Renmin Hospital of Wuhan University, Wuhan, China; ^3^ Department of Urology, Wuhan No. 1 Hospital, Tongji Medical College, Huazhong University of Science and Technology, Wuhan, China

**Keywords:** cell cycle, apoptosis, PI3K/AKT, PTEN, bladder cancer, TSPAN7

## Abstract

The tetraspanin protein superfamily participate in the dynamic regulation of cellular membrane compartments expressed in a variety of tumor types, which may alter the biological properties of cancer cells such as cell development, activation, growth and motility. The role of tetraspanin 7 (TSPAN7) has never been investigated in bladder cancer (BCa). In this study, we aimed to investigate the biological function of TSPAN7 and its therapeutic potential in human BCa. First, *via* reverse transcription and quantitative real-time PCR (qRT-PCR), we observed downregulation of TSPAN7 in BCa tissues samples and cell lines and found that this downregulation was associated with a relatively high tumor stage and tumor grade. Low expression of TSPAN7 was significantly correlated with a much poorer prognosis for BCa patients than was high expression. Immunohistochemistry (IHC) showed that low TSPAN7 expression was a high-risk predictor of BCa patient overall survival. Furthermore, the inhibitory effects of TSPAN7 on the proliferation and migration of BCa cell lines were detected by CCK-8, wound-healing, colony formation and transwell assays *in vitro*. Flow cytometry analysis revealed that TSPAN7 induced BCa cell lines apoptosis and cell cycle arrest. *In vivo*, tumor growth in nude mice bearing tumor xenografts could be obviously affected by overexpression of TSPAN7. Western blotting showed that overexpression of TSPAN7 activated Bax, cleaved caspase-3 and PTEN but inactivated Bcl-2, p-PI3K, and p-AKT to inhibit BCa cell growth *via* the PTEN/PI3K/AKT pathway. Taken together, our study will help identify a potential marker for BCa diagnosis and supply a target molecule for BCa treatment.

## Introduction

Bladder cancer (BCa) is the most common malignancy of the urinary system, with more than 80,000 newly diagnosed cases and almost 18,000 deaths in the USA in 2019 (1). Approximately 70% of all diagnosed cases are non–muscle invasive bladder cancer (NMIBC), whereas the remaining cases are classified as muscle-invasive bladder cancer (MIBC). Despite advancements in the development of novel drugs and surgical treatments, approximately 50% of patients with BCa develop metastatic or recurrent disease within 2 years of diagnosis (2). Patients always require long-term follow-up with cystoscopy and computed tomography (CT) scans in case of relapse. As a result, the management costs of BCa seem to be considerably higher than other cancers (3). The overall survival of BCa patients remains very poor, thus, a better understanding of the molecular mechanisms of bladder carcinogenesis and elucidation of effective methods for predicting the prognosis of BCa are imperative.

Encoded by the TM4SF2 gene on XP114, tetraspanin 7 (TSPAN7) is a member of the tetraspanin protein superfamily of conserved membrane proteins (4–6). Most of the family members are cell-surface proteins that are characterized by the presence of four hydrophobic domains. TSPAN7 was first described as being strongly expressed in T-cell acute lymphoblastic leukemia (ALL) (7). Subsequently, TSPAN7 was found to be expressed in cancer of the stomach, pancreas, liver, esophagus, kidneys, and to be most strongly expressed in the brain (8–11). TSPAN7 mediates signal transduction events that play a role in the regulation of cell development, activation, growth, and motility (6, 12–14). In multiple myeloma (MM) patients, elevated TSPAN7 expression may be associated with better outcomes in up to 50% of patients (15). However, in lung cancer, TSPAN7 promotes migration and proliferation *via* epithelial-to-mesenchymal transition (16). Overall, TSPAN7 expression is associated with carcinogenesis, however, the precise role of TSPAN7 expression in BCa has not been defined.

Herein, by bioinformatics analysis of a dataset from The Cancer Genome Atlas (TCGA-BLCA), combined with fresh BCa and adjacent tissue samples studies, we identified that the downregulation of TSPAN7 expression plays an essential oncogenic role in BCa pathogenesis. In the current study, we first identified that the expression of TSPAN7 was significantly associated with tumor stage and grade in human BCa, and that low TSPAN7 expression was an independent predictive factor of overall survival (OS). Furthermore, overexpression of TSPAN7 exerted negative impacts on cell proliferation, colony formation, apoptosis, migration and invasion both *in vitro* and *in vivo via* the PTEN/PI3K/AKT signaling pathway. Thus, regulation of PTEN/PI3K/AKT signaling *via* TSPAN7 targeting may represent a new therapeutic approach for BCa treatment.

## Materials and Methods

### Bioinformatics Analysis

The mRNA-read scount expression data for 427 bladder urothelial carcinoma patient samples (408 BCa and 19 normal bladder tissue samples) and clinical survival data for 412 patients were downloaded from TCGA-BLCA with “TCGAbiolinks” package in R language. An mRNA expression matrix was made with the raw counts of each RNA in each sample. The “Deseq2” package in R was used to calculate the differential expression of mRNAs between the bladder cancer tissues samples and paracancerous normal specimens. A fold change| >2 and p-value <0.05 were used as the threshold. A volcano plot for the differentially expressed mRNAs was generated with “ggplot2” in R. Survival analysis was performed using these differentially expressed mRNAs with the “ggsurv” package in R with a p-value <0.05 used as the screening threshold. Then, the significant selected mRNAs were functionally analysed by Gene Ontology (GO) enrichment analysis and Kyoto Encyclopedia of Genes and Genomes (KEGG) pathway analysis with a p-value <0.05 set as the statistical threshold, the performing “clusterprofiler” R package was used to screen out significant enrichments in KEGG pathways and GO terms.

### Patients and Tissue Samples

Thirty-four pairs of fresh BCa and adjacent tissue specimens were obtained at the Department of Urology at Renmin Hospital of Wuhan University from March 2019 to December 2019. All specimens were collected by radical resection from patients without a prior history of BCa or adjuvant therapy and harvested after obtaining patients’ written consent. BCa was defined by two pathologists. The tumor stage and grade of all patients were diagnosed according to the 2009 TNM staging system and 2004 World Health Organization grading system, respectively. All patients were under regular follow-up.

### Cell Lines and Cell Culture

The human bladder cancer cell lines 5637, T24, and EJ and human immortalized normal bladder epithelium cell line SV-HUC-1 were kindly provided by the Stem Cell Bank, Chinese Academy of Sciences (Shanghai, China). Identification of the cell lines was conducted at the China Centre for Type Culture Collection (Wuhan, China). 5637, T24, and EJ cells were maintained in RPMI-1640 medium (HyClone, China), and SV-HUC-1 cells were maintained in F-12K medium (HyClone, China) supplemented with 10% fetal bovine serum (FBS) (Gibco, Australia) and 1% penicillin G sodium/streptomycin sulfate. All the cells were grown in a humidified atmosphere consisting of 5% CO_2_ and 95% air at 37°C

### Total RNA Isolation From Bladder Tissue Samples and BCa Cells

Total RNA was extracted from BCa cells and bladder tissue specimens using TRI Reagent (Cat. abs9331-100 ml, Absin, China) according to the manufacturer’s instructions. The reverse transcription process was carried out with the RevertAid RT Reverse Transcription Kit (Cat. K1691, Thermo Scientific, China). Finally, the produced cDNA was stored at -20°C.

### Reverse Transcription and Quantitative Real-Time PCR

A total 20 μl-volume reaction system, which contained 1 μl cDNA, 1 μl of each primer, 10 μl NovoStart^®^ SYBR qPCR SuperMix Plus (Cat.E096-01A, novoprotein, China), and 7 μl DNAse/RNAse-free water, was performed in triplicates. Fold enrichment was calculated with the 2−ΔΔCt method relative to the expression of GAPDH. The primer sequences were listed as follows: TSPAN7: 5`- CTCATCGGAACTGGCACCACTA-3`, 5`- CCTGAAATGCCAGCTACGAGCT-3`; GAPDH: 5`- GTCTCCTCTGACTTCAACAGCG-3`, 5`- ACCACCCTGTTGCTGTAGCCAA-3`. All experiments were conducted in triplicate and repeated three times.

### Immunohistochemistry

For IHC, the procedures of dewaxing and rehydration were similar to those for HE staining. Then, the tissue sections were boiled in citrate buffer (pH 6.0) at 100°C for 15 min. A primary antibody (anti-TSPAN7, 1:50, 18695-1-AP, Proteintech) was added to the tissue sections after blocking with 3.0% hydrogen peroxide (H2O2) for 10 min at room temperature and incubated overnight at 4°C. A secondary antibody was added to the slides and incubated at room temperature for 30 min. Finally, the sections were incubated with DAB chromogen and then counterstained with hematoxylin.

Section assessment was completed by two experimental pathologists who were blinded to clinical outcomes. The scoring of TSPAN7 expression was defined as a score of 0, 1, 2, or 3 according to the staining intensity, and the overall staining score was summarized as low (0, 1) or high (2, 3).

### Transfections and Selection of BCa Cell Lines With Stable Overexpression of TSPAN7

The full sequence of TSPAN7 was inserted into a lentiviral vector to construct a TSPAN7-overexpression plasmid (Vigenebio, China). BCa cells (1×10^5^) were seeded in 6-well plates and grown to approximately 50% confluency. Then, the culture medium was removed, and fresh culture medium containing lentiviral particles carrying TSPAN7 cDNA or a negative control was added according to the manufacturer’s instructions. The cells were cultured in an incubator at 37°C with 5% CO_2_ for 18 h. Next, the culture medium was removed and replaced with fresh medium. After transfection for 72 h, culture medium containing an appropriate concentration of puromycin (Sigma, USA) was added to kill any nontransfected cells. The surviving cell clones were selected and expanded. The lentiviruses were designated pcDNA-TSPAN7. The empty vector was used as a negative control (pcDNA-vector). Western blot and qRT-PCR analyses were used to evaluate infection efficiency.

### Protein Extraction and Western Blot Analysis

Total cellular protein was extracted from BCa cells using a RIPA buffer solution. The samples were placed on ice for 30 min with discontinuous ultrasonic dispersion. The lysates were centrifuged at 12,000 rpm for 15 min at 4°C. The supernatant was harvested, and the protein concentration was detected with a bicinchoninic acid (BCA) assay using bovine serum albumin (BSA) as the standard. The extracted protein samples were denatured at 100°C for 10 min after 25% volume loading buffer was added. Finally, the protein samples were stored at -20°C. A total of 60 µg of protein from each sample was resolved by 8%–12% SDS-PAGE and transferred to PVDF membranes (Millipore, USA), which were blocked with 5% nonfat milk for at least 1 h at room temperature. The membranes were incubated with primary antibodies overnight at 4°C on a table concentrator, followed by secondary antibody incubation for 1 h at room temperature. Bands were detected with a corresponding protein development instrument and quantified with ImageJ software (W S Rasband, ImageJ, NIH).

### CCK-8-Based Cell Viability Assay

To assess cell proliferation, BCa cells were seeded at a density of 2×10^3^ cells/well in 96-well plates and cultured for 24, 48, 72, or 96 h. At each end of the experiment, 10 µl of CCK-8 reagent (CK04, Dojindo, Japan) was added to each well, and the cells were further cultured for 1 h. Absorption values were measured at 450 nm. Cell growth curves were plotted according to the results of each experiment. All experiments were conducted in triplicate and repeated three times.

### Tumor Cell Colony Formation Assay

Tumor cell clonogenicity was assessed with a colony formation assay. Cells were seeded in 6-well plates at 1×10^4^, 1×10^3^, and 1×10^2^ cells/well and grown for 10 days. Visible colonies (≥50 cells) were counted after 4% paraformaldehyde (PFA) fixation and 0.1% crystal violet staining. The experiment was repeated three times.

### Transwell Migration Assay

For transwell migration assays, we used a 24-well plate transwell chamber system (Corning, USA). In the upper chamber, 8×10^4^ cells were suspended in 200 μl of serum-free medium, while 600 μl of 20% FBS medium was added to the lower chamber to induce cell migration. After 72 h, a cotton swab was used to remove any remaining cells in the upper chamber. The cells that migrated to the other side of the membrane were fixed in 4% PFA for 30 min and stained with 0.1% crystal violet for 4 hours. The stained chambers were left to dry and photographed. The experiment was repeated three times.

### Wound-Healing Assay

To assess cell motility, a wound-healing assay was used. Approximately 2–3×10^6^ cells were plated in a 6-well plate. When the cells were 90%–95% confluent, the cell layer was carefully scratched with a sterile tip and washed with PBS three times. The cells were then incubated for 0 h, 12 h, 24 h, and 48 h, and images were acquired. The assays were repeated in triplicate.

### Cell Cycle and Apoptosis

BCa cells were harvested, centrifuged and then washed with cold PBS twice. For cell cycle analysis, cells were resuspended in 1× DNA Staining Solution containing propidium iodide and a permeabilization solution and incubated at 37°C for 30 min in the dark. The cell cycle distribution of each sample was analyzed by flow cytometry analysis. For cell apoptosis analysis, cells were stained with the Annexin-V FITC Apoptosis Detection Kit I (BD Biosciences, USA) according to the kit protocol and analyzed by flow cytometry analysis.

### TUNEL Assay

In brief, Prepare paraffin sections → dewaxing and hydration → cell transparency → add TUNEL reaction solution (TUNEL, Roche Applied Science, Germany) → add Converter -POD→ react with substrate DAB to develop color → count and take photos with optical microscope.

### Xenograft Mouse Model

Specific pathogen-free (SPF) male BALB/c-nude mice (4 weeks old) were purchased from Beijing HFK Bioscience Co., Ltd. (Beijing, China). After a week of adaption at the laboratory animal facility of Renmin Hospital of Wuhan University, we randomly assigned mice to the control group and the test group. For a subcutaneous tumor growth assay, 1×10^6^ pcDNA-TSPAN7 or pcDNA-NC T24 cells diluted in 0.2 ml of serum-free medium were subcutaneously injected into 5-week-old BALB/c-nude mice. After 5 weeks, the mice were sacrificed, all of the xenotransplanted tumors were dissected, and tumor weight and tumor size were measured with a Vernier caliper (tumor volume = length×width^2^×0.5 mm^3^). The tumors were fixed in 4% PFA and subsequently analyzed by IHC staining.

### Statistical Analysis

The 23.0 SPSS software package was used for all statistical analyses. The significance of differences was compared using the χ^2^ test and Student’s t test. Overall survival was estimated by the Kaplan-Meier method, and differences in survival between two groups were analyzed by the log-rank test. For univariate and multivariate analyses, the Cox proportional hazards regression model was used. A two-sided P value < 0.05 was considered statistically significant.

## Results

### Sixteen Key Marker Genes Were Selected by Bioinformatic Analysis

**Figure 1:**
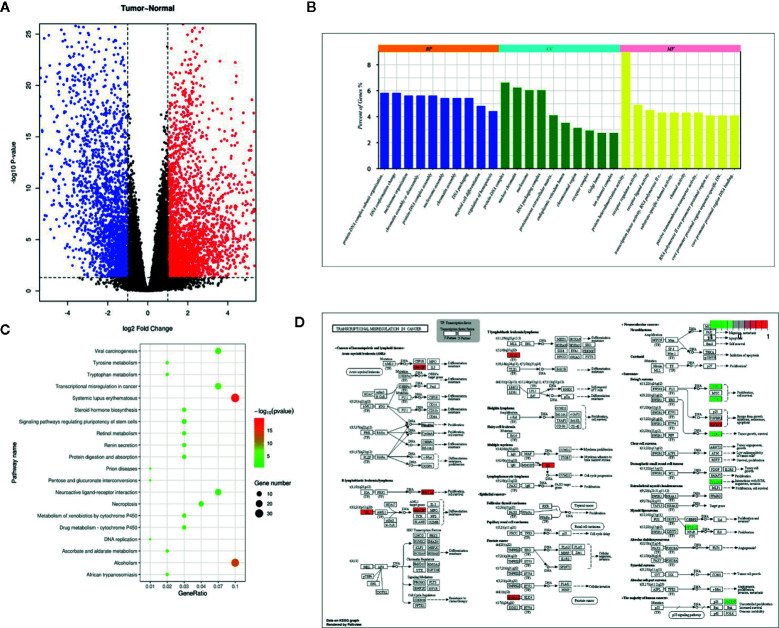
Differentially expressed genes in TCGA dataset, and pathway enrichment of TSPAN7. **(A)** Volcano plot visualizing the all differentially expressed genes in TCGA dataset, **(B)** GO enrichment, **(C)** KEGG pathway, and **(D)** Transcriptional misregulation in cancer.

Through differential expression analysis of 19858 mRNAs between BCa and paracancerous normal specimens, 4943 significantly differentially expressed mRNAs including 2786 upregulated and 2157 downregulated mRNAs were obtained (**Figure 1A**). Herein, GAGE12D, CT45A5, GAGE2B, FGB, CT45A1, GAGE2D, GAGE1, and GAGE2A were upregulated with >10000-fold changes, and FAM180B, KCNB1, MYH11, PI16, MYOC, SYNM, GPR112, OSTN, MYH2, and GLP2R were downregulated with >40-fold changes in the BCa tissue samples compared to the normal bladder tissue samples. These differentially expressed RNAs were used to perform survival analysis exploring the effects of these mRNAs on the survival prognosis of BCa patients. Significantly (p-value <0.05), 596 mRNAs associated with a favorable or poor survival prognosis in bladder cancer were identified. The 596 mRNAs were functionally enriched in GO terms spanning the biological process (BP), molecular function (MF) and cellular component (CC) categories, and KEGG pathways, which showed relatively significant terms, such as DNA conformation changes, protein-DNA complex, receptor regulator activity and transcription factor activity (**Figures 1B–D**). Transcriptional misregulation in a cancer pathway was identified to be significant and found to involve 16 genes: HIST1H3D, HIST1H3B, HIST1H3F, HIST2H3D, CSF2, TLX3, HIST1H3A, HIST1H3E, IGFBP3, ETV7, WNT16, SLC45A3, GADD45A, ID2, TSPAN7, NFKBIZ, and IGF1.

### TSPAN7 Downregulation in BCa Tissue Specimens and Cell Lines

**Figure 2:**
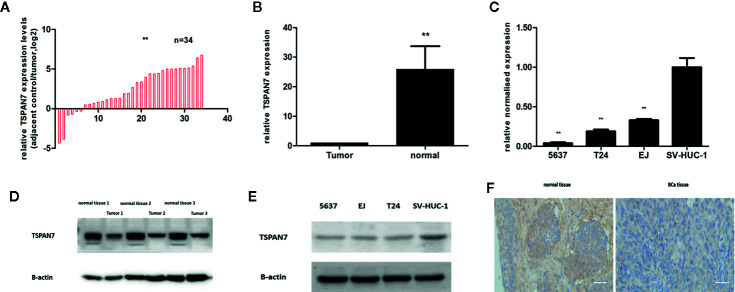
TSPAN7 was downregulation in BCa patients and cell lines. **(A, B)** TSPAN7 mRNA expression in BCa tissue samples was lower than in normal tissue. **(C)** TSPAN7 mRNA was significantly downregulation in BCa cell lines (T24, 5637, EJ) compared with the normal bladder epithelial cell line (SV-HUC-1). **(D)** In Western blot, TSPAN7 showed a higher expression in normal tissue. **(E)** In Western blot, TSPAN7 showed a higher expression in normal bladder epithelial cell line. **(F)** Immunohistochemistry (IHC) showed the protein level of TSPAN7 was increased in normal bladder tissue. Scale bars, 100μm. **p<0.01.

We first assessed TSPAN7 mRNA expression in BCa tissue samples compared to normal tissue samples (**Figures 2A, B**) and in cell lines (**Figure 2C**). qRT-PCR showed that TSPAN7 mRNA was significantly higher in the normal bladder tissue specimens than in the BCa tissue specimens. The same result was found in Western blot (**Figures 2D, E**) of the BCa samples and cell lines. IHC staining results (**Figure 2F**) showed that the protein level of TSPAN7 was increased in normal bladder tissue specimens.

### Associations of TSPAN7 Expression With The Clinicopathological Features and Survival of Bladder Cancer Patients

**Figure 3:**
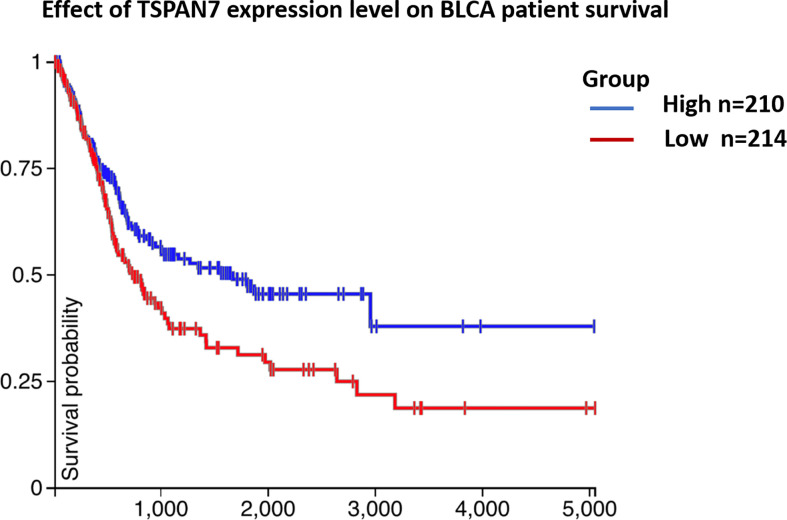
(**Table 1**) Association between TSPAN7 expression and clinicopathological features of human bladder cancer. (**Figure 3**) TSPAN7 expression was significantly associated with poor overall survival from UCSC.

As shown in **Table 1**, TSPAN7 expression was associated with tumor stage (p=0.01) and tumor grade (p=0.03) in BCa. However, no relationships were found between TSPAN7 expression and other clinical features, such as patient sex (p=0.68), age (p=0.41), tumor size (p=0.67), and tumor multiplicity (p=0.87), lymphnodes status (p=0.53). We then analyzed data from UCSC for patient overall survival and found that reduced TSPAN7 expression was significantly associated with poor overall survival (**Figure 3**).

**Table 1:** Correlation between TSPAN7 expression and clinical features of patients.

Variable	Groups	Total	Low	High	p
**Gender**	Male	25	21	4	0.68
	Female	9	7	2	
**Age (years)**	≥60	22	19	3	0.41
	<60	12	9	3	
**Tumor size (cm)**	≥	20	16	4	0.67
	<3	14	12	2	
**Multiplicity of tumor**	Single	18	15	3	0.87
	Multiple	16	13	3	
**Tumor grade**	PUNLMP, low grade	8	3	5	0.01
	High grade	26	25	1	
**Tumor stage**	Ta, T1	10	6	4	0.03
	T2–T4	24	22	2	
**Lymphnodes**	Negative	26	22	4	0.53
	Positive	8	6	2	
**Distant metastasis**	Absent	34	28	6	
	Present	0			

### TSPAN7 Is Negatively Correlated With BCa Cell Proliferation, Viability, and Migration *In Vitro*

**Figure 4:**
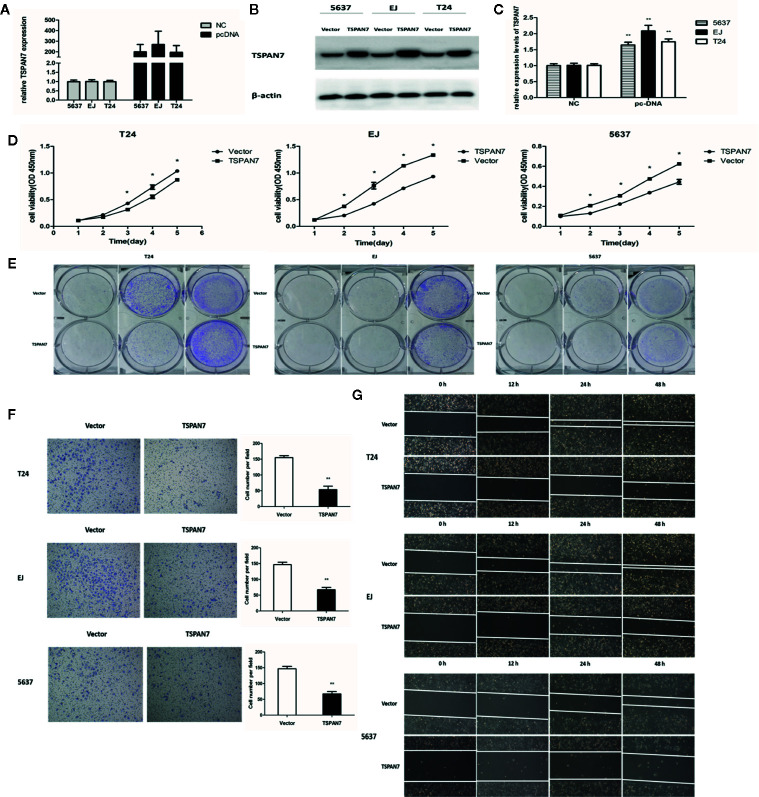
Overexpression of TSPAN7 repressed BCa cell proliferation and migration. **(A)** Verification of TSPAN7 overexpression efficacy at the mRNA level in T24, EJ, 5637 cells. **(B, C)** Verification of TSPAN7 overexpression efficacy at the protein level in T24, EJ, 5637 cells. CCK-8 assays **(D)** and colony formation assays **(E)** showed that TSPAN7 overexpression decreased the proliferation capacity. Transwell invasion **(F)** and wound-healing assays **(G)** showed that TSPAN7 overexpression attenuated cell migration ability *P < 0.05; **P < 0.01.

Our current data demonstrated that TSPAN7 expression was reduced in BCa tissue and cell lines and that TSPAN7 downregulation was associated with poor overall survival. We further assessed whether changes in TSPAN7 expression could affect BCa cell malignant behaviors. qRT-PCR (**Figure 4A**) and Western blot (**Figures 4B, C**) data confirmed that the expression of TSPAN7 was upregulated in pcDNA-TSPAN7 groups compared with pcDNA-vector groups. We then found that overexpression of TSPAN7 inhibited cell proliferation (**Figure 4D**). BCa cell colony formation assays showed that TSPAN7 overexpression reduced the number of BCa cell colonies (**Figure 4E**). Transwell invasion (**Figure 4F**) and wound-healing assays (**Figure 4G**) verified that TSPAN7 overexpression inhibited BCa cell invasion.

### Effects of TSPAN7 on BCa Cell Apoptosis *In Vitro*

**Figure 5:**
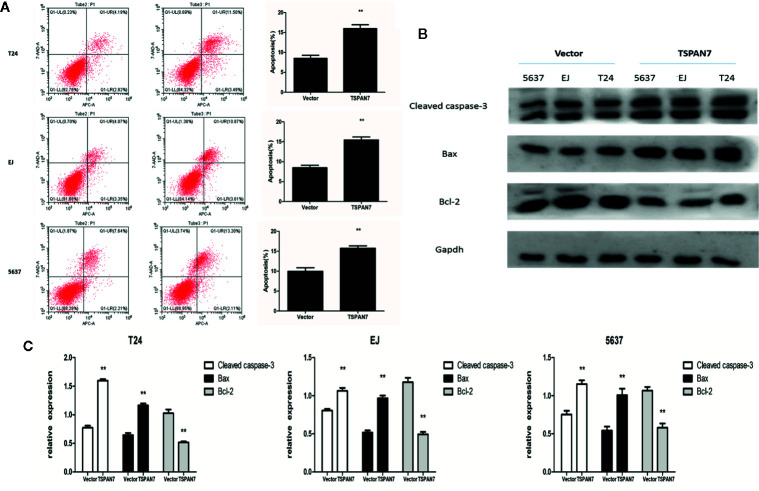
TSPAN7 overexpression promotes the apoptosis of BCa cell. **(A)** Quantitative flow cytometry measurements of apoptosis in T24, EJ, 5637 cells. **(B, C)** TSPAN7 overexpression upregulated the expression of cleaved caspase-3 and Bax whereas that of Bcl-2 was downregulated. **p<0.01 vs. the control group. All the above data are the mean ± SD from an average of three experiments.

Next, we determined the effects of TSPAN7 overexpression on BCa cell apoptosis. We found that the percentage of apoptotic cells was significantly higher in pcDNA-TSPAN7 groups than in pcDNA-vector groups (**Figure 5A**). Western blot data further showed that the expression of cleaved caspase-3 and Bax was upregulated, whereas that of Bcl-2 was downregulated in the pcDNA-TSPAN7 groups (**Figures 5B, C**).

### Effects of TSPAN7 on BCa Cell Cycle Arrest *In Vitro*

**Figure 6:**
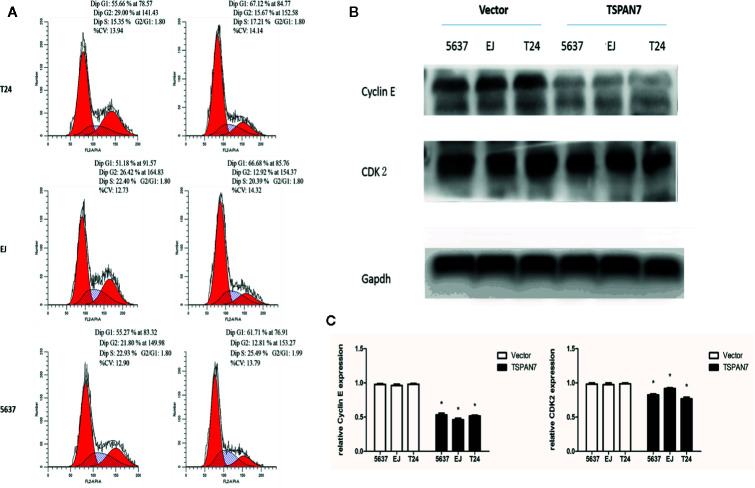
**(A)** TSPAN7 overexpression induced cell cycle arrest at the G1/S phase. **(B)** CDK2 and cyclin E expression was downregulated in pcDNA-TSPAN7 groups. **(C)** Densitometry analysis of western blots showed quantitation of Cyclin E and CDK2 levels. *p<0.05.

Moreover, TSPAN7 overexpression in 5637, EJ, and T24 cells increased the proportion of cells in the G1 phase compared to control expression (**Figure 6A**). Western blot data showed that CDK2 and cyclin E expression was downregulated in pcDNA-TSPAN7 groups compared to pcDNA-vector groups (**Figures 6B, C**). These findings suggested that TSPAN7 overexpression induced cell cycle arrest in the G1 phase of the cell cycle.

### TSPAN7 Inhibits Proliferation in BCa Cell Lines Through The PTEN/PI3K/AKT Pathway

**Figure 7:**
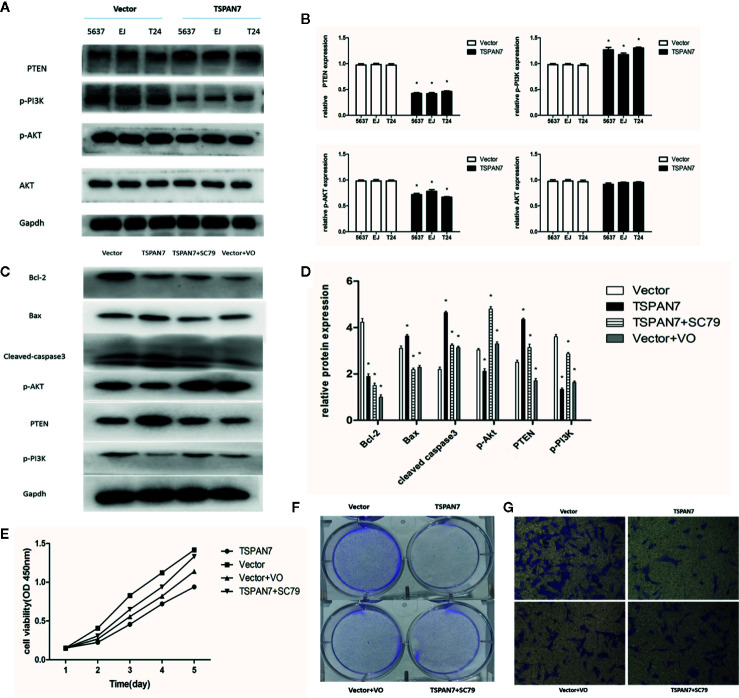
TSPAN7 inhibits proliferation in BCa cell lines *via* PTEN-PI3K/AKT pathways. **(A, B)** TSPAN7 overexpression in BCa cells downregulated the expression of p-PI3K and p-AKT, and upregulated the expression of PTEN. **(C, D)** In Western blot, AKT agonist SC79 could reverse the effect of TSPAN7 overexpression on T24 cells and PTEN inhibitor VO-Ohpic trihydrate caused the similar effect on pcDNA-vector T24 cells. **(E–G)** In the presence of SC79, the proliferation, migration and invasion of pcDNA-TSPAN7 T24 cells were clearly elevated and inhibition of PTEN in T24 cells distinctly decreased cell growth, migration and invasion. *P < 0.05 vs. the corresponding NC cells. All the above data are the mean ± SD from an average of three experiments.

Transactivation of PI3K/AKT can cause different biological activities, such as inflammation, immunity, cell growth, tumorigenesis, and apoptosis (17–19). In this study, we measured PI3K/AKT expression and activity in pcDNA-TSPAN7 and pcDNA-vector groups. We found that TSPAN7 overexpression in BCa cells downregulated the expression of p-PI3K and p-AKT, and upregulated the expression of PTEN, whereas pcDNA-vector did not impact these proteins (**Figures 7A, B**).

Then, we assessed whether the AKT agonist SC79 could reverse the effect of TSPAN7 overexpression on T24 cells. We also used PTEN inhibitor VO-Ohpic trihydrate to verify whether it caused the similar effect on pcDNA-vector T24 cells. The p-AKT levels in T24 cells were significantly elevated after SC79 treatment, and PTEN expression was markedly suppressed after VO-Ohpic trihydrate treatment (**Figures 7C, D**). Compared with no treatment, treatment of cells with SC79 or VO-Ohpic trihydrate dramatically produced opposite effects on the levels of these proteins. These findings indicated that SC79 partly reversed the inhibitory effect of TSPAN7 overexpression on T24 cells and that VO-Ohpic trihydrate showed an effect similar to that of TSPAN7 overexpression. To investigate the role of the PTEN/PI3K/AKT pathway in TSPAN7-mediated cell proliferation, migration and invasion, we performed rescue experiments also. In the presence of SC79, the proliferation, migration, and invasion of pcDNA-TSPAN7 T24 cells were clearly elevated. Similarly, inhibition of PTEN in T24 cells distinctly decreased cell growth, migration and invasion (**Figures 7E–G**). Altogether, these results confirmed that TSPAN7 inhibited the PTEN/PI3K/AKT pathway upstream of AKT and downregulated PTEN/PI3K/AKT pathway activation.

### Overexpression of TSPAN7 Suppresses BCa Cell Growth *In Vivo*

**Figure 8:**
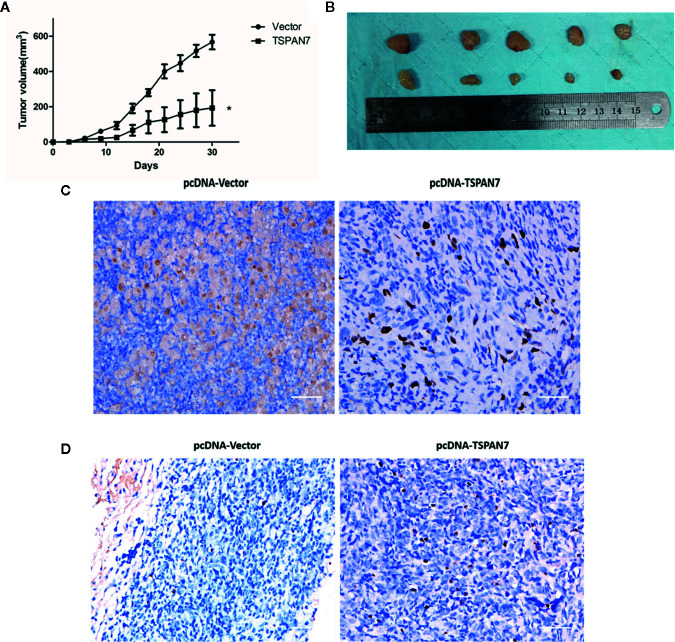
Anti-tumor effects of *in vivo*. **(A)** Mean tumor volume at each time point. **(B)** Morphology of the subcutaneous implanted tumor. **(C)** Immunohistochemistry (IHC) was performed to detect the protein of Ki67 in the tumor tissue. **(D)** A TUNEL assay was performed to detect the apoptotic cells in the tumor tissue. *P < 0.05 vs. the control. All the above data are the mean ± SD from an average of three experiments.

To confirm the inhibitory effects of TSPAN7 *in vivo*, we subcutaneously injected pcDNA-TSPAN7 or pcDNA-vector T24 cells into nude mice. We found significant differences in T24 cell xenograft formation, growth and weight between the two groups (**Figures 8A, B**). The size of tumor xenografts was larger in the pcDNA-vector T24 cell group than in the pcDNA-TSPAN7 groups. IHC showed that Ki67 expression was significantly downregulated in pcDNA-TSPAN7 T24 tumors compared to pcDNA-vector tumors (**Figure 8C**). Next, TUNEL staining validated that apoptotic cell numbers were increased in the pcDNA-TSPAN7 groups compared with the pcDNA-vector groups (**Figure 8D**). These results suggest that TSPAN7 suppresses tumor growth *in vivo*.

## Discussion

In the present study, we showed that the expression of TSPAN7 in normal bladder tissue and cells was significantly higher than that in BCa tissue and cells. Furthermore, high expression of TSPAN7 was negatively correlated with a high T stage and tumor grade in BCa. The survival of patients with high expression of TSPAN7 was superior to that of those with low expression. Moreover, TSPAN7 overexpression inhibited BCa cell proliferation, cell cycle progression, invasion, and apoptosis.

TSPAN7 is a member of the transmembrane 4 superfamily, also called the tetraspanin family, which includes proteins characterized by four transmembrane domains, with one short and one large extracellular loop (20). Previous studies have found that in cerebellar granule cells, TSPAN7 promotes axonal branching, and the size of TSPAN7 clusters is increased by downregulation of IGSF3 expression, which might be at the center of a new signaling pathway controlling brain development (21). In oral tongue squamous cell carcinoma, differential methylation of TSPAN7 was found to be predictive of certain clinical and epidemiologic parameters (22). There is also research suggesting that TSPAN7 plays an important role in the cytoskeletal organization required for the bone-resorbing function of osteoclasts by regulating signaling to Src, Pyk2, and microtubules (23). Lee SA disclosed a previously uncharacterized role for TSPAN7 in the regulation of the expression and functional activity of the dopamine D2 (DRD2) receptor, which was implicated in multiple neurologic and psychiatric disorders by postendocytic trafficking (24). In clear cell renal cell carcinoma (CCRCC), relatively high TSPAN7 expression in primary tumor cells is not associated with patient outcomes (25). However, increased TSPAN7 expression in CCRCC lung metastases is associated with prolonged metastasis-free survival (11). To the best of our knowledge, this is the first study to identify elevated TSPAN7 expression in BCa. Our study provides the first genetic evidence that TSPAN7 plays a critical role in BCa tumorigenesis. Analyses of clinicopathological features showed that TSPAN7 was an independent prognostic factor of BCa that was significantly correlated with T stage and tumor grade, and low expression of TSPAN7 predicted a poor prognosis (OS) in BCa patients. According to our transcriptomic analysis, the mRNA expression of TSPAN7 was strongly downregulated in BCa tissue samples versus adjacent tissue samples, in accordance with the results from our TCGA database and qRT-PCR analyses. Consistent with the TCGA database analysis, the downregulation of TSPAN7 expression at both the transcriptional and translational levels in tumor specimens predicted high malignancy and a poor prognosis in BCa patients. Our results showed that overexpression of TSPAN7 inhibited BCa cell growth, migration and invasion *in vitro* and *in vivo*.

Furthermore, our findings revealed that overexpression of TSPAN7 could induce BCa cell apoptosis with caspase 3 cleavage and elevate the Bax/Bcl-2 ratio, indicating a potential role for TSPAN7 in facilitating apoptosis. The intrinsic apoptotic pathway (mitochondria-dependent) activated in response to different stress conditions is mediated by intracellular signals that converge at the mitochondrial level (26). The Bcl-2 family regulates both proapoptotic and antiapoptotic pathways controlling MOMP alteration (27). Therefore, Bcl-2 family proteins serve as an “apoptotic switch” by mediating permeabilization of the mitochondrial membrane (28). The balance and interactions among Bcl-2 family members can determine whether a cell survives or undergoes apoptosis. While antiapoptotic proteins regulate apoptosis by blocking the mitochondrial release of cytochrome c, proapoptotic proteins act by promoting this release. Activation of the Bcl-2 family (Bax and Bak) neutralizes the antiapoptotic proteins Bcl-2 and Bcl-xL, leading to disruption of mitochondrial membrane outer membrane permeability (MOMP) so that proteins such as cytochrome-*c*, which plays a crucial role in activating mitochondrial-dependent death, are released into the cytosol (29). Then, cytochrome-c triggers the formation of apoptosomes, which recruit initiator pro-caspase-9 to the caspase recruitment domain (CARD), resulting in autoactivation and proteolysis (30). Then, the process activates downstream executors, such as caspase-3, caspase-6 and caspase-7, for cleavage of cellular substrates, leading to apoptotic cell death (31).

Next, a series of gain-of-function assays was performed to elucidate the regulatory functions of TSPAN7 in BCa cells. Overexpression of TSPAN7 demonstrated a marked inhibitory effect on BCa cells by reducing proliferation, attenuating cell migration and inducing G1/S cell cycle arrest. Furthermore, Western blot analysis indicated that overexpression of TSPAN7 interfered with G0/G1 phase-related proteins, such as CCNA1/2, CCND1, and CDK2/4. Cancer often represents a pathological manifestation of uncontrolled cell division and cell cycle dysregulation. In mammalian cells, the G1-to-S phase transition requires the formation of cyclins D and E and activation of the cyclin D-CDK4/6 and cyclin E-CDK2 complexes (32). These proteins phosphorylate and inactivate Rb to release E2F, which mediates transcriptional activity. Then, the cell cycle will enter the S phase (33). The G2-to-M phase transition requires the activation of the cyclin B-CDK1 complex *via* the dephosphorylation of CDK1 (34). CDK2 promotes S phase initiation *via* the formation of functional cyclin A and cyclin E complexes (35). Upregulation of CDK2 expression can be found in various solid tumors and is closely related to the development of tumors (36). In the present study, TSPAN7 was found to have a close relationship with CDK2, which binds to cyclin E to initiate the G1-to-S phase transition. This is accord with other studies (37, 38).

Phosphatase and tensin homolog (PTEN) is a tumor suppressor gene that was discovered in 1997 (39). It has been proven that the protein encoded by PTEN has protein phosphatase and lipid phosphatase activities, which can regulate a complex network dependent on phosphatase or nonphosphatase activity to affect cell biological functions (40–42). The frequent loss of heterozygosity, the inverse correlation between PTEN dose and tumorigenicity and the variety of PTEN regulatory mechanisms suggest that altering PTEN levels in cells may affect tumor progression, including that of thyroid, breast, and prostate cancer (41, 43–46). PTEN antagonizes growth factor-stimulated PI3K/AKT signaling by converting PIP3 to PIP2. PTEN dephosphorylates phosphatidylinositol 3,4,5-triphosphate (PIP3) and attenuates the activity of class I phosphatidylinositol 3-kinase (PI3K), which mediates survival factor signaling through PI3K effectors, such as AKT and mTOR (47). A previous study also indicated that PTEN is a tumor suppressor in the progression of cancers that functions by negatively regulating the PI3K/AKT signaling pathway (48). It has also been reported that, the activation of PI3K/AKT signaling, as a significant cancer-promoting pathway, blocks cellular apoptosis and accelerates cell proliferation *via* the activation of PTEN (49). Our present study was in accordance with the results of the aforementioned studies. We found elevated expression levels of PTEN and cleaved Caspase-3 but reduced expression levels of p-PI3K and p-AKT in the pcDNA-TSPAN7 group compared to the control group.

Finally, we established a xenograft tumor model using nude mice and demonstrated that TSPAN7 inhibited tumorigenesis *in vivo*.

In conclusion, we have shown, for the first time, the tumor-inhibiting effects of TSPAN7 on human BCa. TSPAN7 acts as a biomarker to predict the survival of BCa patients and the malignancy of tumors. TSPAN7 could be an oncogene that promotes apoptosis and inhibits tumor growth and cell cycle progression in BCa *via* the regulation of multiple key components of the PTEN/PI3K/AKT pathway. Specifically, it would be worthwhile to investigate whether restoring TSPAN7 expression can be a novel therapeutic strategy for BCa.

## Data Availability Statement

The raw data of this article will be made available by the authors, without undue reservation.

## Ethics Statement

The studies involving human participants were reviewed and approved by Ethics Committee of Renmin Hospital of Wuhan University. The patients/participants provided their written informed consent to participate in this study. The animal study was reviewed and approved by the Ethics Committee of Renmin Hospital of Wuhan University.

## Author Contributions

XL and LW conceptualized the study. SL contributed to the data curation. MP conducted the formal analysis. XY, TX, and LW investigated the study. YD contributed to the methodology. KY and SZ conducted the project administration. TB and JH conducted the visualization. XY wrote the original draft. XY and SL wrote, reviewed, and edited the manuscript. All authors contributed to the article and approved the submitted version.

## Funding

This study was supported by the National Natural Science Foundation of China (No. 81972408 and 82000639), the Application and Basic Research Project of Wuhan City (No. 2018060401011321), and the Innovation Project of Medical School of Wuhan University (TFZZ2018017).

## Conflict of Interest

The authors declare that the research was conducted in the absence of any commercial or financial relationships that could be construed as a potential conflict of interest.
